# Eye Movements as an Index of Pathologist Visual Expertise: A Pilot Study

**DOI:** 10.1371/journal.pone.0103447

**Published:** 2014-08-01

**Authors:** Tad T. Brunyé, Patricia A. Carney, Kimberly H. Allison, Linda G. Shapiro, Donald L. Weaver, Joann G. Elmore

**Affiliations:** 1 Department of Psychology, Tufts University, Medford, Massachusetts, United States of America; 2 Department of Family Medicine, Oregon Health and Science University, Portland, Oregon, United States of America; 3 Department of Pathology, Stanford University School of Medicine, Palo Alto, California, United States of America; 4 Department of Computer Science and Engineering, University of Washington, Seattle, Washington, United States of America; 5 Department of Pathology, University of Vermont and Vermont Cancer Center, Burlington, Vermont, United States of America; 6 Department of Medicine, University of Washington, Seattle, Washington, United States of America; University Medical Centre Utrecht, Netherlands

## Abstract

A pilot study examined the extent to which eye movements occurring during interpretation of digitized breast biopsy whole slide images (WSI) can distinguish novice interpreters from experts, informing assessments of competency progression during training and across the physician-learning continuum. A pathologist with fellowship training in breast pathology interpreted digital WSI of breast tissue and marked the region of highest diagnostic relevance (dROI). These same images were then evaluated using computer vision techniques to identify visually salient regions of interest (vROI) without diagnostic relevance. A non-invasive eye tracking system recorded pathologists’ (N = 7) visual behavior during image interpretation, and we measured differential viewing of vROIs versus dROIs according to their level of expertise. Pathologists with relatively low expertise in interpreting breast pathology were more likely to fixate on, and subsequently return to, diagnostically irrelevant vROIs relative to experts. Repeatedly fixating on the distracting vROI showed limited value in predicting diagnostic failure. These preliminary results suggest that eye movements occurring during digital slide interpretation can characterize expertise development by demonstrating differential attraction to diagnostically relevant versus visually distracting image regions. These results carry both theoretical implications and potential for monitoring and evaluating student progress and providing automated feedback and scanning guidance in educational settings.

## Introduction

Developing visual expertise is fundamental to the accuracy of physicians’ interpretations of optical images, such as those integral to pathology, radiology, and dermatology practice. However, little is known about how visual expertise develops during and after training, which perceptual and cognitive mechanisms are responsible for expertise development, and how educators can evaluate its progress over time [1]–[3]. These issues are especially important given the Accreditation Council on Graduate Medical Education’s (ACGME) focus on competency development, which is part of their Next Accreditation System [4]. This new system will require evidence that residents demonstrate competence in examining and assessing surgical pathology specimens, which must be submitted to the ACGME as part of the Milestones project. Medical educators in residency training programs are currently working on how best to characterize learners’ competence. More research on objective measures to accomplish this is needed to inform educators and educational researchers. Specific to pathology interpretation, research in the cognitive sciences has demonstrated that experts move their eyes differently compared to novices, [1], [5]–[7]. Experts are able to quickly identify suspicious regions at low magnification and then spontaneously identify diagnostically relevant features for later fixation and interpretation [2], [5]. Novices tend to overtly fixate on multiple image features that are both relevant and irrelevant to the ultimate diagnosis.

Several theories have been proposed to account for how learners acquire specialized visual expertise. One proposes that novices consider all image features prior to generating differential hypotheses and arriving at a final diagnosis [8]. This detailed search process results in a focus on both relevant *and* irrelevant features, the formation of multiple competing diagnostic hypotheses and, sometimes, diagnostic errors [9]. As novices develop expertise, they reduce the number of image features they consider and move more quickly to identify and interpret visual features that guide accurate diagnoses [10]. For a range of visual concepts, expertise can only develop if the learner is exposed to and subsequently diagnoses a sufficient range of abnormalities. This leads to the development of visual accuracy of particular diagnoses. This memory based, top-down (i.e., under cognitive control) guidance of visual attention is considered a critical component of visual expertise development [11].

A number of studies have demonstrated that novices take more time interpreting images compared to experts, they fixate more often and move their eyes more often between fixations, while also spending more time fixating on diagnostically irrelevant image features [2], [12]. Very little work has attempted to characterize how novices identify salient visual features needed to rule in or rule out different diagnoses, which assists in developing diagnostic accuracy [13]. In one such study, Krupinsky and colleagues showed that experts viewed a digitized breast biopsy for approximately 4.5 seconds whereas residents viewed for 7.1 seconds; residents also tended to fixate nearly 3 times as frequently on a given slide relative to experts. In that study, however, slide interpretation was performed at a fixed low zoom level (see also [14]), and no analyses were conducted to examine whether novices were more attracted to salient but diagnostically uninformative visual features. Advances in visual and cognitive sciences have resulted in highly validated quantitative approaches for analyzing images for visually salient features and predicting eye movements towards these features [15]. In general, these analytic methods involve generating saliency maps that highlight visual regions of interest with salient background orientations, colors, and intensities that are visually salient but possibly unrelated to specific diagnoses (vROI) [16]. Saliency maps have proven highly reliable at predicting visual attention and are considered reflective of bottom-up (i.e., feature-based) visual processing.

In this study, we tested whether saliency maps, as a measurement tool, can aid in discriminating between novice and expert pathologists’ viewing behavior while interpreting digitized breast specimens. Specifically, we hypothesized that novices and experts would display differential viewing of the vROI versus dROI, and relative attractions to the vROI would predict diagnostic errors; indeed increased attraction to diagnostically uninformative regions may be associated with a failure to identify diagnostic features [17]. Support for such a hypothesis would carry both theoretical and practical implications. Theoretically, this would help in defining the visual mechanisms responsible for novice’s increased interpretation times, including a greater number of fixations, and greater attention toward diagnostically irrelevant areas. Practically, the development of visual expertise is fundamental to graduate and post-graduate pathology education and training. Because no quantitative methods or metrics exist for evaluating the development of visual expertise independent from diagnostic accuracy, we propose that dissociating attention toward visually salient versus diagnostically relevant image regions may prove valuable in monitoring progress in visual expertise development and potentially informing the design of educational curricula.

## Methods

### 2.1.1 Participants

We recruited seven physicians with a range of experience interpreting breast pathology. We included three residents with limited breast pathology experience, two faculty members specializing in dermatopathology and general anatomic pathology, and two physician faculty members who specialize in breast pathology. Participants provided written informed consent. All materials and study activities were reviewed and approved by Institutional Review Boards at the University of Washington (#41467) and Tufts University (#1109018).

### 2.1.2 Materials & Equipment

#### 2.1.2.1 Image Test Set

A test set of 10 digital whole slide image (WSI) breast specimens was selected from a larger test set developed as part of an ongoing National Cancer Institute (NCI) funded breast pathology study [18]. Specimens were obtained from cancer registries participating in the HIPAA compliant [19] Breast Cancer Surveillance Consortium (BCSC) in Vermont and New Hampshire [20]. Women from whom the samples were obtained provided prior consent for their archived tissue samples to be used for research and were ≥40 years of age at the time of breast biopsy. Each specimen was hematoxylin and eosin (H&E) stained, and associated with a single “gold standard” diagnosis established using a modified Delphi approach [21] with three expert breast pathologists’ independent interpretations and subsequent consensus meetings. The ten cases were chosen using a stratified sampling technique to ensure we included a range of diagnoses: two proliferative, one atypical lobular hyperplasia (ALH), one lobular carcinoma in situ (LCIS), two ductal carcinoma in situ (DCIS; nuclear grade 2), and two invasive breast cancer specimens. For each specimen, an expert breast pathologist (coauthor DLW) identified a single region of interest most representative of the diagnosis; we will refer to this region as the diagnostic region of interest (dROI). Slides were scanned into digital TIFF format using an iScan Coreo Au digital slide scanner [22] at 40x magnification.

#### 2.1.2.2 Visual Saliency Algorithm

**Figure 1:**
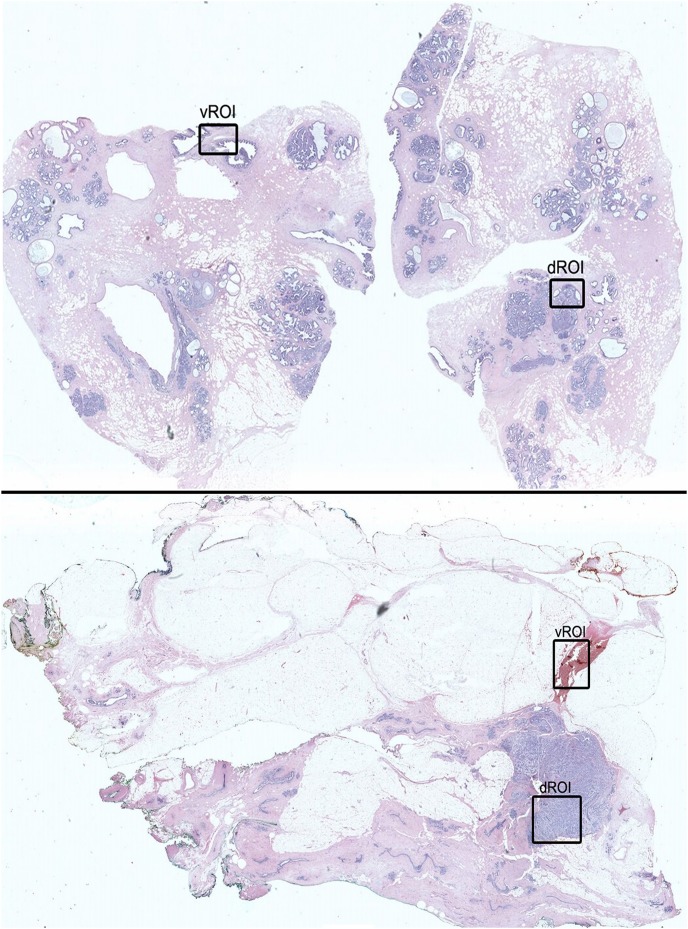
Regions of interest derived from expert diagnosis (dROI) and visual saliency algorithm (vROI). A single dROI was defined per image, based on expert consensus; the dROI thus indicates the DCIS (top panel) and Invasive (bottom panel) region of highest diagnostic value. A single vROI was defined per image, based on a winner-takes-all approach using only the highest ranking visually salient region.

Each of the 10 digital whole slide breast images was evaluated by computing maps that encode the saliency of visual features on each image. We used a well-validated algorithm for computing the saliency maps [16], where the algorithm proceeds through 4 processing stages: 1) It performs linear image filtering at 8 scales: color (RGBY), intensity, and orientation (0,45,90,135°), which extracts low-level visual features using Gaussian pyramids; 2), It computes differences between center and surround areas for each visual feature, which results in generating feature and conspicuity maps; 3) It performs linear summing of conspicuity maps into a single saliency map; and 4) The final version of this map is computed using neural network modeling, and corresponds to stimulus features that are most likely to capture visual attention. Saliency maps were generated by using freely-available MATLAB scripts [23]. The result of this process is that the saliency map predicts where naïve observers will look on the image by identifying the region with the most visually salient features of interest; we refer to this region as the visual region of interest (vROI; Figure 1).

#### 2.1.2.3 Eye Tracker & Computer

Eye movements were monitored using SensoMotoric Instruments (SMI; Boston, MA) non-invasive remote eye-tracking device (RED). This infrared camera-based system tracks monocular eye movements at 60 Hz. Calibration for each participant was performed using the integrated SMI iView software, with a nine-point process attaining <0.5° error in visual angle. The eye tracker was mounted to the bottom of a 19″ LCD monitor, which operated at 1280×1024 pixels resolution.

#### 2.1.2.4 Digital Image Viewer

A custom digital slide viewer was used to display images in a navigable viewport. The viewer was developed to allow zooming (1–60x; using either buttons or mouse wheel) and panning (using mouse click/drag actions) while maintaining full-slide resolution. The viewport logged all pathologist navigation behavior, to include current view position within the image coordinate system and zoom level.

### 2.1.3 Data Collection Procedures

Following consent, participants completed a brief calibration session, which involved watching a dot move between nine points on the screen, repeating itself until gaze tracking error fell below 0.5°. Participants then completed a brief practice session while viewing a benign breast specimen to become familiar with the image viewer. Each of the 10 images was then presented one at a time in random order on the computer monitor, using the digital image viewer at full screen. Participants were allowed to take as much time as necessary to examine each image, after which they indicated a final diagnosis on a standard histological assessment form.

### 2.1.4 Data Processing & Analysis

Raw eye tracking data are output as Cartesian coordinate positions made by the eye over time. This raw data stream was pre-processed using conventional methods [24] to parse into fixations (momentary eye movement restrictions exceeding a temporal threshold). Fixations are considered a primary indicator of overt visual attention. Because our image viewer allowed for both panning in *x, y* coordinate space, and zooming in *z* space, we converted gaze coordinates (recorded in the monitor coordinate system) to pan- and zoom-contingent image space (recorded in the viewport coordinate system) over time. This process allowed us to use a common spatial reference system for both eye tracking and viewport data.

During the analysis process, the 10 specimen images were grouped by diagnoses with similar clinical treatments, producing three overarching categories: benign/atypia (five images), carcinoma in situ (three images), and invasive (two images). Participants were divided into three groups [25]–[27]: 1) *Novices* were comprised of current residents with limited breast pathology experience (n = 3); 2) *Intermediates* were comprised of faculty members specializing in dermatopathology and general anatomic pathology (n = 2), and 3) *Experts* were comprised of faculty who specialized in breast pathology (n = 2).

Analyses focused on seven dependent measures. The first two aimed at replicating a greater overall number of fixations, and a lengthier overall interpretation time for novices versus experts (for a review, see Reingold & Sheridan, 2011). The remaining five measures examined our primary hypotheses where each measure was used to compare novice versus expert data. First, we assessed the total number of eye fixations falling within the coordinate region of the dROI versus vROI. Second, we compared the total amount of time spent fixated in dROIs versus vROIs. Third, we examined the precedence of fixations falling within the dROI versus vROI. Fourth, we compared the number of regressions back to the dROI versus vROI; regressions (or “re-visits”) occur when fixations leave and then return to a particular region.

Finally, we examined whether the number of eye fixations falling within dROI and vROI predicted diagnostic accuracy. In all analyses comparing fixation counts we calculated standardized residuals (z-scores) relative to expected values, using 2.0 as a threshold for significance [28]. In analyses comparing interpretation times we performed one-way analyses of variance (ANOVAs) within each diagnostic category. As detailed in our Results, initial analyses demonstrated highly similar data patterns according to diagnostic category; to increase statistical power and ease interpretation, analyses assessing dROI and vROI fixation data as a function of pathologist expertise use data collapsed across diagnoses.

## Results

### 3.1.1 Accuracy & Eye Movements

Overall diagnostic accuracy within each category did not vary according to expertise, though it did vary across the three diagnostic categories, with lower accuracy (*M* = .48) among cases of carcinoma in situ relative to benign/atypia (*M* = .83) and invasive (*M* = .93). Because accuracy data followed a non-normal distribution, we confirmed this via nonparametric chi-square test, χ^2^(2) = 8.0, *p*<.05.

Within each of the three diagnostic categories (benign/atypia, carcinoma in situ, invasive), the number of fixations varied according to participant expertise (Table 1). Within all three diagnostic categories, Novices showed a greater frequency of fixations relative to expected values (*R*_std_ = Benign/Atypia: 17.65; Carcinoma in Situ: 13.97; Invasive: 5.72). In contrast, Experts showed a lower frequency of fixations relative to expected values in all categories except Invasive (*R*_std_ = Benign/Atypia: 3.23; Carcinoma in Situ: 10.67, Invasive: 1.78). Intermediates generally patterned similarly with Experts, with a lower frequency of fixations relative to expected values (*R*_std_ = Benign/Atypia: 18.38, Carcinoma in Situ: 6.44, Invasive: 8.78).

**Table 1:** Mean (and standard error) number of fixations and viewing time (in seconds) as a function of diagnostic category and expertise group.

Participant Expertise	Diagnostic Category		
	Benign/Atypia	Carcinoma in Situ	Invasive
***Number of Fixations***			
**Novices**	238 (34.2)	233 (63.3)	97.2 (15)
**Intermediates**	99.9 (35.7)	137.5 (71.5)	38.3 (16.3)
**Experts**	163.7 (69.3)	114.8 (30.5)	84.5 (43)
**Chi-square χ^2^(df = 2)**	659.8 (*p*<.01)	350.2 (*p*<.01)	112.9 (*p*<.01)
***Mean Viewing Time***			
**Novices**	113.3 (19.9)	102.7 (26.7)	67.5 (18.3)
**Intermediates**	47.2 (20.9)	64.7 (34.2)	16 (7.2)
**Experts**	90.9 (44.3)	67.1 (21.6)	48.9 (31.4)
**ANOVA ** ***F*** **(df = 3)**	1.5 (*p* = .33)	.62 (*p* = .58)	1.6 (*p* = .32)

For number of fixations, chi-square test statistics, derived from testing total frequency counts, are provided for each of the three diagnostic categories. For mean viewing time, test statistics are derived from one-way analyses of variance (ANOVA) comparing the three expertise groups.

Within each of the three diagnostic categories, Novices consistently viewed the images for longer durations than Experts or Intermediates (Table 1). No differences, however, reached significance (*p_min_* = .32).

### 3.1.2 dROI versus vROI Analyses

Fixations in the dROI were similar between Novices and Experts, though Intermediates showed a greater number of fixations in this region (Table 2; *R*_std_ = 3.37). As hypothesized, Novices showed nearly twice as many fixations in the distracting vROI relative to Experts. Specifically, Novices showed a greater number of vROI fixations, and Experts fewer vROI fixations, relative to expected values (*R*_std_ = 2.14, 2.43, respectively).

**Table 2:** Total number of fixations falling within the diagnostic (dROI) and visual (vROI) regions of interest as a function of expertise group.

	Mean # of Fixations per Image		Mean Time Fixated (sec)		Fixation Precedence		Mean # of vROI Regressions
	dROI	vROI	dROI	vROI	dROI	vROI	
**Novices**	9.7	2.8	2.11	0.65	3	14	1.40
**Intermediates**	13.1	1.9	2.56	0.39	2	6	0.63
**Experts**	9.7	1.6	2.04	0.33	7	2	0.52
**Statistic**	?^2^(2) = 16.9 (*p*<.01)	?^2^(2) = 10.6 (*p*<.01)	*F*(3) = .19 (*p* = .84)	*F*(3) = 2.4 (*p* = .21)	?^2^(2) = 9.8 (*p*<.01)		?^2^(2) = 11.2 (*p*<.01)

Test statistics tests provided within each measure are derived from either chi-square tests (on total frequency counts), or one-way analyses of variance (ANOVA), comparing the three expertise groups.

We found subtle differences in mean time fixated in the dROI and vROI, and neither region showed significant differences according to level of expertise. Though not reaching statistical significance, Novices did spend nearly twice as much time, on average, fixated in the distracting vROI relative to Experts. We also found that Novices showed a vROI precedence (vROI preceding dROI fixations), and Experts showed the opposite pattern (dROI preceding vROI fixations). Thus, Novices were more likely to fixate on the distracting vROI prior to fixating on the diagnostically relevant dROI, and Experts were more likely to do the opposite.

We also considered the sequences of fixations that returned to the vROI after having left the region for at least a single fixation. This allowed us to consider repeated visual interest in distracting vROI. As expected, Novices were nearly three times as likely to re-visit the distracting vROI relative to Experts, and over twice as likely relative to Intermediates. We found a greater than expected number of vROI regressions among Novices (*R*_std_ = 2.46), and fewer vROI regressions among Experts (*R*_std_ = 2.06), relative to expected values (Intermediate did not deviate from expected, *R*_std_ = 0.95).

We conducted two simple linear regression tests to assess whether the mean number of fixations falling within the dROI versus vROI across participants might positively or negatively predict diagnostic accuracy. The first regression examined dROI fixations and found no predictive value of the number of dROI fixations, *F*(6)<.01, *p* = .94, β = −.001, *R* ^2^<.01. The second regression examined vROI fixations and found a negative relationship between the number of vROI fixation and diagnostic accuracy, though this pattern did not reach significance, *F*(6) = .49, *p* = .51, β = −.043, *R* ^2^ = .09. Thus, there is some directional suggestion that attending to the distracting vROI may predict diagnostic failures, but continuing work must explore this question with larger participant samples.

## Discussion

Data from this pilot study support some but not all of our original hypotheses. First, we found that novices showed a greater overall number of eye fixations and longer viewing times compared to experts. This finding was stronger when cases were associated with inherently higher diagnostic difficulty (i.e., LCIS, DCIS), with Novices spending between 24.6 and 53.1% more time than Experts. Though this pattern did not reach traditional significance levels (perhaps due to our limited sample size), it provides quantitative support for earlier work suggesting that Novices require substantially longer viewing times relative to Experts, even when ultimately arriving at an identical diagnosis.

Second, we also hypothesized that Novices would show stronger evidence of spontaneous visual attraction to distracting non-diagnostically relevant regions, and indeed we found that Novice pathologists showed a greater number of fixations on the distracting vROI relative to Experts and Intermediates. We did not find this same pattern for the diagnostically relevant regions (dROI). Novice pathologists also showed a longer, though non-significant, amount of average time fixated on the distracting vROI (but not dROI) relative to Experts and Intermediates. When pathologists fixated on both the vROI and dROI, we found that Novices tended to fixate on the vROI first, then the dROI. Experts tended to not look at the vROI; though when they did, it typically occurred after having already viewed the dROI. Finally, we examined how many times each pathologist re-visited the distracting vROI after having already fixated in it. Here, we found that Novice pathologists tended to re-visit the distracting region nearly three times as often as Experts.

Together our measures provide unique preliminary evidence that Novices may be more vulnerable to visual distraction by features that lack diagnostic relevance, and that this is a dynamic process that recurs throughout the viewing experience. From an educational theory [8], [29] perspective, this supports the notion that Novices consider both relevant and irrelevant image features, while Expert viewers quickly and holistically parse an image into features relevant to diagnosis. Our result is also consistent with research that indicates novices over-rely on salient perceptual features at the expense of top-down attentional guidance toward diagnostically relevant features [29]. Experts, by definition, have viewed a relatively broad range of abnormalities and thus have developed rich visual concepts or “cognitive schema” [30] that accurately capture diagnoses [31]. These visual concepts are used to efficiently guide visual attention to diagnostically relevant image regions. In this manner, through training and experience, physicians become more attuned to diagnostic features and are less vulnerable to distracting image regions. Enhanced specific domain knowledge on behalf of experts, along with potentially different motivational or effort levels and varied clinical contexts, all independently and interactively guide diagnostic efficiency and accuracy [32], [33].

Novices’ increased viewing of irrelevant image regions may be driven by bottom-up (i.e., solely based on image features) attention but then activates a mechanism that sequentially rules out alternate diagnostic possibilities. Theories of visual attention suggest that detecting the absence of a particular feature is more difficult than detecting its presence, leading to lengthier search times [34]. Similarly, Nodine and Mello-Thoms [35] proposed that experts follow a *detect-then-search* process involving rapid detection of diagnostically relevant features followed by a brief search to ensure the absence of other relevant features. In contrast, Novices follow a *search-then-detect* process, which involves extensive visual search and perception of multiple irrelevant features, then ultimately detection of diagnostically relevant features. These search and “rule-out” mechanisms are likely responsible for the increased number of fixations and increased viewing times among Novice pathologists.

Findings from this study suggest that eye movements may ultimately be used to evaluate the development of visual expertise in fields such as pathology, especially when distinguishing between visual attention to diagnostically relevant versus visually distracting irrelevant regions. Kundel and Nodine [31] proposed that education and training progressively alters the process of medical image interpretation by modifying students’ visual search strategies and ultimate interpretations. As such, they proposed that a critical development in expertise is the ability to disambiguate diagnostically relevant visual features from irrelevant visual noise. We provide empirical support for this concept, adding a possible metric to educators’ and mentors’ toolkit for evaluating expertise development through education and training.

This pilot study has several limitations. We had a limited sample size (n = 7) that may limit statistical power and, ultimately, interpretation. However, our effects, when they emerged, were of substantial magnitude and showed highly reliable numerical patterns across each of our measures. It is possible that Type II errors (i.e., incorrectly failing to reject the null) may have occurred in our viewing time measures, which we aim to further investigate in future data collection. Moreover, all of our participants were sampled from a relatively restricted set of pathologists within a single academic institution. Continuing research should attempt to replicate our results using sampling strategies that include a diverse population across institutions and regions. In addition, to make this type of research relevant to educators, including residents at all levels of training by program year would be helpful. Though residency faculty must now characterize competency of levels of learners and report these biannually to the ACGME in the Next Accreditation System [4], it is unclear at this time that the use of eye movement assessments will contribute to assessments of diagnostic accuracy, and whether it will be financially or logistically feasible for such assessments. We also suggest that continuing research aims to better quantify pathologist expertise with digital image viewers; though our pathologists all likely held low familiarity with digital image viewing, variation in experience with this medium might influence the efficiency of navigating the image in search of diagnostic features. Finally, it is possible that our experiment may have unintentionally influenced pathologists’ viewing behavior or diagnostic criteria. While we recognize this possibility, we note that our images were diverse and our image viewer interface was designed to emulate several visual and manual features of commonly used digital image software. We also note that diagnostic accuracy was overall high, and all participants appeared to take the task seriously and remained engaged throughout the session.

In conclusion, we provide the first evidence that visual attraction to salient but diagnostically irrelevant image features may underlie the inherently different viewing behavior of Novices versus Experts while interpreting digitized breast specimens. This finding supports and extends extant educational theories, suggesting that Novices frequently and repeatedly fixate on distracting image regions, potentially using this time to identify histopathologic features that aid in ruling out alternate diagnoses. The present results also suggest that attentional biases toward irrelevant yet visually salient image features may prove valuable in educational settings, as it suggests promising avenues for monitoring and evaluating individual student progress, and providing real-time or post-hoc feedback and guidance regarding visual scanning behavior. Innovative technologies such as eye tracking afford such possibilities and, along with empirical approaches to educational system design, may ultimately hold great potential for increasing pathologist diagnostic efficiency and accuracy. Because all competency assessments require established benchmarks, the quantitative outcomes derived from eye tracking may prove valuable complements to traditional metrics of pathologist development, such as in-service examinations and subjective evaluation [36]. Though our findings are early and preliminary in nature, they represent exciting new possibilities for both theory and practice in medical education.
